# Building Blocks of Hybrid Perovskites: A Photoluminescence Study of Lead‐Iodide Solution Species

**DOI:** 10.1002/cphc.202000479

**Published:** 2020-09-30

**Authors:** Oleksandra Shargaieva, Lena Kuske, Jörg Rappich, Eva Unger, Norbert H. Nickel

**Affiliations:** ^1^ Young Investigator Group „Hybrid Materials Formation and Scaling“ Helmholtz-Zentrum Berlin für Materialien und Energie GmbH Kekuléstr. 5 12489 Berlin Germany; ^2^ Interdisziplinäres Zentrum für Materialwissenschaften Martin-Luther Universität Heinrich-Damerow-Str. 4 06120 Halle Germany; ^3^ Institute Silicon Photovoltaics Helmholtz-Zentrum Berlin für Materialien und Energie GmbH Kekuléstr. 5 12489 Berlin Germany

**Keywords:** coordination chemistry, hybrid perovskites, ligand-to-metal charge transfer, optical properties of solution species, polyiodide plumbates

## Abstract

In this work, we present a detailed investigation of the optical properties of hybrid perovskite building blocks, [PbI_2+n_]^n−^, that form in solutions of CH_3_NH_3_PbI_3_ and PbI_2_. The absorbance, photoluminescence (PL) and photoluminescence excitation (PLE) spectra of CH_3_NH_3_PbI_3_ and PbI_2_ solutions were measured in various solvents and a broad concentration range. Both CH_3_NH_3_PbI_3_ and PbI_2_ solutions exhibit absorption features attributed to [PbI_3_]^1−^ and [PbI_4_]^2−^ complexes. Therefore, we propose a new mechanism for the formation of polymeric polyiodide plumbates in solutions of pristine PbI_2_. For the first time, we show that the [PbI_2+n_]^n−^ species in both solutions of CH_3_NH_3_PbI_3_ and PbI_2_ exhibit a photoluminescence peak at about 760 nm. Our findings prove that the spectroscopic properties of both CH_3_NH_3_PbI_3_ and PbI_2_ solutions are dominated by coordination complexes between Pb^2+^ and I^−^. Finally, the impact of these complexes on the properties of solid‐state perovskite semiconductors is discussed in terms of defect formation and defect tolerance.

## Graphical abstract

**The optical properties** of hybrid perovskite „building blocks” – polyiodide plumbates ([PbI_2+n_]^n−^) – formed in solutions of CH_3_NH_3_PbI_3_ and lead iodide are studied. This study provides a deep insight into the fundamental driving forces for [PbI_2+n_]^n−^ formation in both solutions and a better understanding of the role of [PbI_2+n_]^n−^ solution complexes in „defect tolerance” of hybrid perovskites.

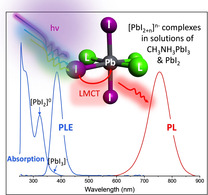

## 1 Introduction

Hybrid perovskites exhibit outstanding optoelectronic properties such as high absorption coefficients^1^, ^2^ and large charge‐carrier diffusion length,^3^, ^4^, ^5^, ^6^, ^7^ which are comparable to those of conventional inorganic semiconductors. These materials can be easily deposited from a solution,^8^, ^9^, ^10^ similarly to organic semiconductors. Recent reports have demonstrated power conversion efficiencies of roll‐to‐roll deposited photovoltaic devices of about 16.1 % on active areas of 802 cm^2^, placing hybrid perovskites on the verge of commercialization.^11^ Further improvement of the device performance is possible through rational process optimization to enable large‐area homogeneous and high‐crystalline quality perovskite layers. To enable the development of reproducible processing conditions, the chemical nature and properties of precursor complexes in solution, their nucleation and growth into solid perovskite semiconductors need to be understood.

Several reports indicated the benefit of solvent engineering in metal‐halide perovskite processing.^12^, ^13^, ^14^, ^15^, ^16^ Using solvent mixtures such as dimethylsulfoxide (DMSO) and N‐dimethylformamide (DMF) or DMSO and gamma‐butyrolactone (GBL) enables processing of homogeneous, highly crystalline, and uniform films.^13^, ^17^, ^18^, ^19^, ^20^ It is believed that film formation is driven by the formation of a Lewis base adduct of lead‐halide precursors and aprotic polar solvent molecules such as DMSO.^21^ The formation of crystalline intermediate phases incorporating solvent molecules such as (MA)_2_(DMF)_2_Pb_2_I_6_ has been experimentally verified.^22^, ^23^

Stamplecoskie et al.^24^ have shown the formation of polyiodide plumbate complexes such as [PbI_3_]^1−^ and [PbI_4_]^2−^ in lead iodide solutions in the presence of large excess iodide ions that were stabilized by coordination of solvent molecules. These lead‐halide‐solvent complexes act as building blocks for perovskite crystals and layers. Hence, understanding the formation mechanism of these building blocks and their properties is of key scientific interest.^25^ The presence of such plumbate complexes in the solid‐state semiconductor might cause defect states which diminish the optoelectronic properties of the semiconductor.^26^, ^27^, ^28^ Therefore, understanding the optoelectronic properties of the precursor solutions may provide deeper insight into the mechanisms that govern the formation and crystallization processes of halide perovskites and unravel the cause for the widely reported high defect tolerance of this material.^29^

In this work, we present a study of the optical properties of polyiodide plumbate complexes, [PbI_2+n_]^n−^. The formation of [PbI_3_]^1−^ and [PbI_4_]^2−^ complexes is demonstrated in solutions of CH_3_NH_3_PbI_3_ and pure PbI_2_ independent of the presence of the organic cation (CH_3_NH_3_I) and nature of the solvent. In addition, we here suggest the formation of polymeric polyiodide plumbates in solutions with high concentration of PbI_2_. For the first time, we report the photoluminescence (PL) and photoluminescence excitation (PLE) spectra of polyiodide plumbate solution species. Both solutions of CH_3_NH_3_PbI_3_ and PbI_2_ exhibit an emission peak at 760 nm. The emission of [PbI_2+n_]^n−^ complexes exhibits a large Stokes shift compared to absorption bands, while it occurs at a similar wavelength as solid CH_3_NH_3_PbI_3_ thin films. This may have important implications on the defect tolerance of metal‐halide perovskite semiconductors.

## 2 Results and Discussion

**Figure 1:**
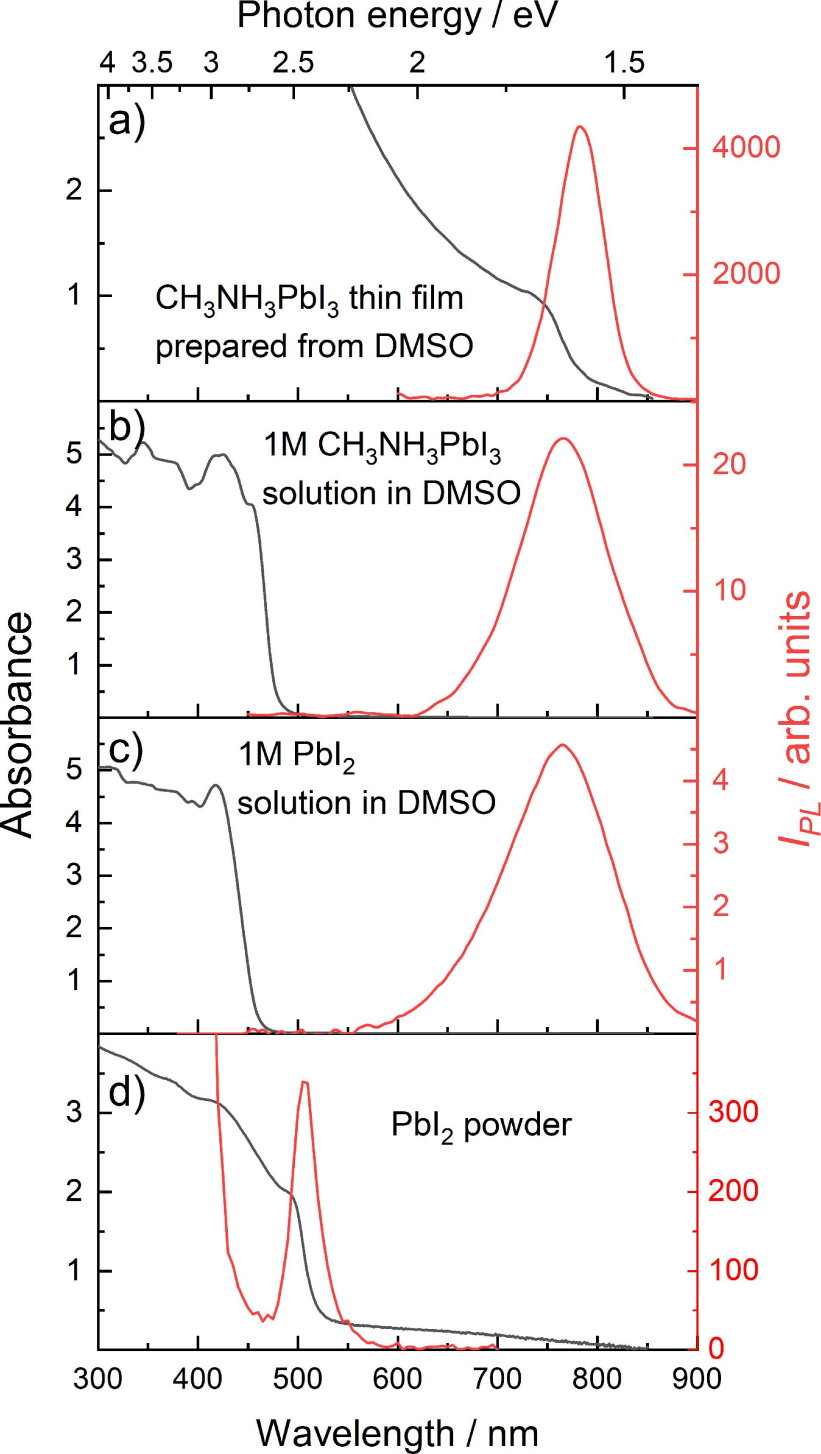
Photoluminescence (PL) (red lines) and absorbance spectra (black lines) of (a) a CH_3_NH_3_PbI_3_ thin film prepared from dimethyl sulfoxide (DMSO). (b) and (c) show the absorbance and PL of a 1 molar CH_3_NH_3_PbI_3_ and PbI_2_ precursor solution in DMSO, respectively. The PL and absorbance spectra of a PbI_2_ powder are shown in (d). For the PL measurements, a pulsed dye laser with an excitation wavelength of *λ*_ex_=367 nm, a pulse width of 0.5 nm, and a pulse energy of 10–20 nJ was used for (a). For the data shown in (b), (c) and (d) a pulse energy of 10–20 μJ and a repetition rate of 10 Hz was applied.

The absorption (black lines) and photoluminescence (PL) spectra (red lines) of CH_3_NH_3_PbI_3_ and PbI_2_ thin films and corresponding precursor solutions are shown in Figure 1. The absorption spectrum of a solid CH_3_NH_3_PbI_3_ thin film exhibits an onset at a wavelength of 765 nm (1.62 eV), which is in good agreement with values from the literature.^30^ The PL spectrum of the same specimen exhibits a maximum at a wavelength of about 780 nm that corresponds to 1.59 eV.

The absorption and PL spectra of the corresponding precursor solution are presented in Figure 1 (b). The 1 molar solution of CH_3_NH_3_PbI_3_ in DMSO exhibits an absorption onset at 465 nm, which is consistent with the previously reported values.^27^, ^31^ However, the emission spectrum of the same solution shows a pronounced red‐shift of the PL peak to 765 nm. This behavior is unexpected and indicates that solution species formed between perovskite precursors CH_3_NH_3_I and PbI_2_ exhibit a large Stokes shift and similar electronic properties as solid‐state CH_3_NH_3_PbI_3_. To test this hypothesis and to exclude the influence of CH_3_NH_3_I, a 1 molar solution of PbI_2_ in DMSO was measured as a reference. The obtained PL and absorption spectra are depicted in Figure 1 (c). The solution exhibits a small blue‐shift of the absorption onset to a wavelength of 440 nm compared to the solution of CH_3_NH_3_PbI_3_ in DMSO. However, the solution of PbI_2_ in DMSO shows the PL emission peak at the same wavelength as the CH_3_NH_3_PbI_3_ precursor solution (*λ*=765 nm). The significance of this observation becomes even more apparent when comparing the absorption and emission spectra of the PbI_2_ solution with a crystalline PbI_2_ powder sample shown in Figure 1 (d). Since PbI_2_ is a yellow‐colored semiconductor with the bandgap of 2.45 eV (*λ=*505 nm),^32^ the presence of the PL peak at *λ=*765 nm in solution clearly indicates that the observed spectral features are originating from similar lead‐iodide‐solvent species, which must be present in both precursor solutions. Most importantly, the formation of these species is independent of the presence of the methylammonium cation.

**Figure 2:**
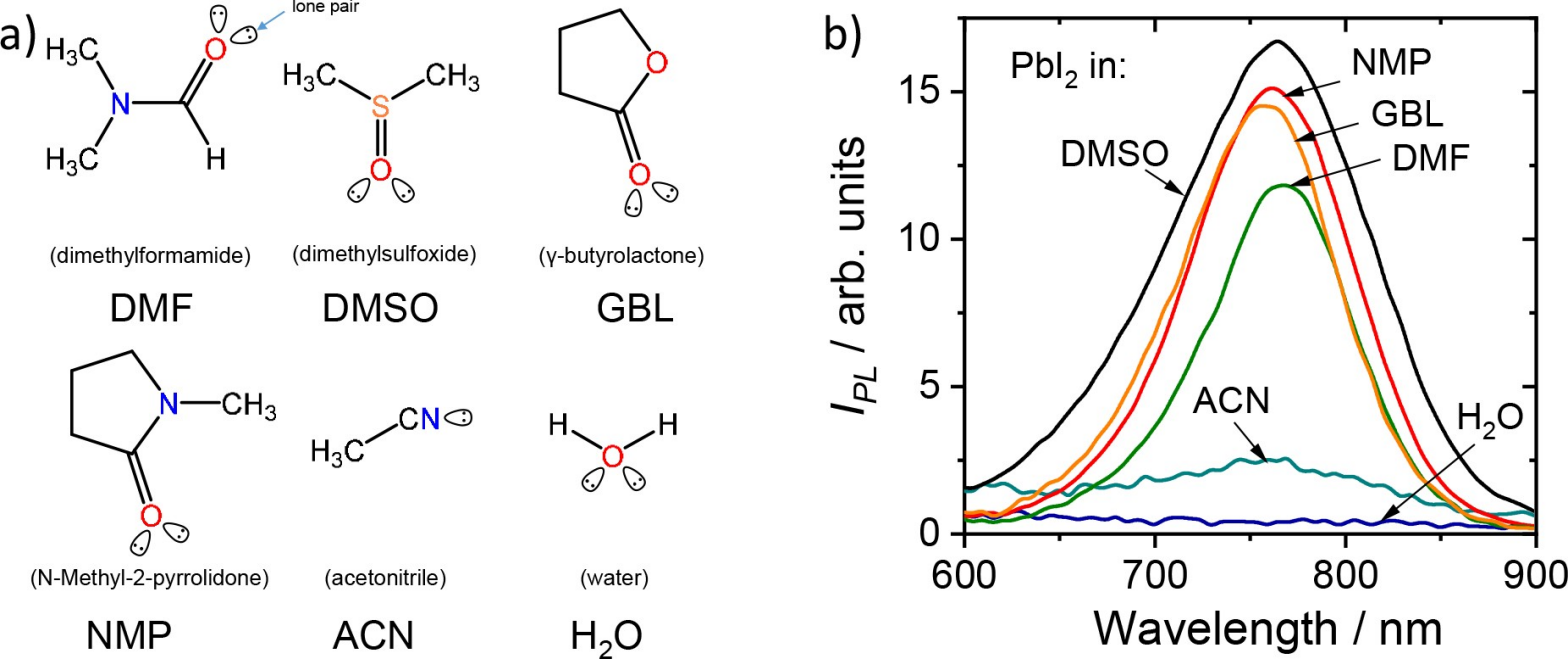
The chemical structures of the used solvents are depicted in (a). In (b) the photoluminescence spectra of PbI_2_ solutions in the solvents: γ‐butyrolactone (GBL), acetonitrile (ACN), N‐Methyl‐2‐pyrrolidone (NMP), dimethyl sulfoxide (DMSO), dimethylformamide (DMF), and water (H_2_O) are shown. The concentration of PbI_2_ in NMP, DMSO, and DMF amounted to 0.1 M. For GBL, ACN, and water solutions the concentration was 2 mM.

One of the possible lead‐iodide‐solvent interactions could be attributed to the formation of an s^2^ metal‐localized complex of Pb^2+^‐L_6_ structure, where L corresponds to a ligand. For the PbI_2_ solution in DMSO, possible ligands are solvent molecules and/or iodide ions, I^−^. The coordination of solvent molecules in such systems often occurs through donor‐acceptor bonding where lone electron pairs of O or S atoms of aprotic polar solvent molecules act as a donor of electrons and empty 6p orbitals of Pb^2+^ ions act as an acceptor. Commonly, the optical transitions of such complexes are strongly affected by the nature of the ligands. Therefore, to distinguish between ligands responsible for the PL of these complexes, the solutions of PbI_2_ in solvents with different polarity and chemical structure were investigated. N‐Methyl‐2‐pyrrolidone (NMP), dimethylformamide (DMF), γ‐butyrolactone (GBL), acetonitrile (ACN), and water were chosen as solvents for the preparation of the solutions. The chemical structures of the corresponding solvents are depicted in Figure 2 (a). Notably, all solvents previously reported for the preparation of perovskite precursor solutions are Lewis bases with at least one lone electron pair located at the oxygen (O), nitrogen (N), or sulfur (S) atoms. This is required for the formation of donor‐acceptor bonds in coordination compounds.

Figure 2 (b) shows the PL spectra of the PbI_2_ solutions with different solvents. Intriguingly, the 0.1 M solutions of PbI_2_ in aprotic solvents such as DMSO, DMF, and NMP exhibit the PL peaks in close proximity to the PL peak of crystalline CH_3_NH_3_PbI_3_ with only slight solvatochromic effects caused by a change in the polarity of the solvents. Due to the limited solubility of lead iodide in γ‐butyrolactone, acetonitrile, and water, the concentration of these solutions was 2 mM. The solution of PbI_2_ in acetonitrile and GBL showed a modest intensity peak at 760 nm, while the aqueous solution of PbI_2_ did not show any emission at 760 nm. Most likely, this is due to either the formation of non‐emissive hydrates or the poor solubility of PbI_2_ in water.^33^ Importantly, none of the PL spectra did exhibit a peak at 505 nm, indicating the absence of species reminiscent of crystalline PbI_2_ (see Figure 1 (d) for comparison). Since the position of the PL peak at 760 nm was altered only modestly by the change in structure and polarity of the solvent, it is plausible that the observed optical transitions originate from coordinate bonding between lead and iodide ions only. Thus, the emissive properties of CH_3_NH_3_PbI_3_ and PbI_2_ solutions are determined by the formation of [PbI_2+n_]^n−^ complexes.^24^

**Figure 3:**
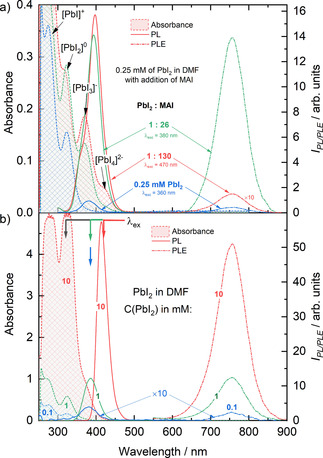
(a) Absorbance spectra (shaded areas), photoluminescence emission (PL) (dash‐dotted lines) and excitation (PLE) (solid lines) spectra of the 0.25 mM solution of PbI_2_ in DMF (blue lines), with the addition of 0.2 M solution of MAI in DMF to the PbI_2_ solution in molar ratio PbI_2_ : MAI of 1 : 26 (green lines) and 1 : 130 (red lines). (b) Absorbance spectra (shaded areas), PL (dash‐dotted lines), and PLE spectra (solid lines) of the PbI_2_ in DMF solution, where the concentration of PbI_2_ amounted to 0.1, 1, and 10 mM. The position of excitation wavelengths of photoluminescence emission spectra is indicated by arrows of the corresponding color. The PLE spectra were detected at *λ*=755 nm.

To elaborate on the emissive properties of polyiodide plumbates [PbI_2+n_]^n−^, we investigated the optical properties of the PbI_2_ and CH_3_NH_3_PbI_3_ solutions. It has been shown previously that [PbI_3_]^1−^ and [PbI_4_]^2−^ species can be generated by the addition of iodine ions (I^−^) to PbI_2_ solution.^24^ The absorption, photoluminescence (PL), and photoluminescence excitation (PLE) spectra of PbI_2_ solution as a function of the concentration of added methylammonium iodide (MAI) are shown in Figure 3 (a) and Figure S1. A low concentration (0.25 mM) solution of PbI_2_ in DMF (blue lines) exhibits absorption bands at 280 and 320 nm that were previously attributed to the lead‐iodide species [PbI_1_]^1+^ and [PbI_2_]^0^, respectively.^24^, ^27^ Excitation of the solution with *λ_exc_*=300 or 320 nm that correspond to the absorption bands of these species, does not yield a significant PL, while excitation with *λ_exc_*=360 nm that corresponds to the absorption band of the [PbI_3_]^1−^ species, leads to the photoluminescence peak at *λ*=760 nm (blue dash‐dotted line). The PLE spectrum of the 0.25 mM PbI_2_ solution exhibits a maximum intensity at 376 nm, matching the position of the absorption bands of [PbI_3_]^1−^ species in solution (Figure 3 (a), S2 (a)).

When adding methylammonium iodide (MAI) in a stoichiometric ratio of 1 : 26 relative to the 0.25 mM PbI_2_ solution, an increase of the intensity of absorption band at 320 nm and an appearance of new bands at 370 nm and 420 nm was observed. The appearance of the bands at 370 nm and 420 nm corresponds to the formation of polyiodide plumbate species [PbI_3_]^1−^ and [PbI_4_]^2−^. This is consistent with previous reports.^24^, ^27^ The deliberate generation of [PbI_3_]^1−^ and [PbI_4_]^2−^ species in solution resulted in a pronounced increase of the intensity of the PL peak at 760 nm by a factor of 30 (green dash‐dotted line). Adding more MAI leads to a further increase in the intensity of the absorption band at 420 nm. This increase indicates a shift of the equilibrium towards the formation of [PbI_4_]^2−^ species in the solution. Similarly to absorption, the PLE spectrum of the solution with 1 : 130 of PbI_2_ : MAI exhibits an increase in the PLE intensity in the long‐wavelength part of the spectrum due to the increase in [PbI_4_]^2−^ concentration (Figure 3 (a), S2 (b)). The appearance of the shoulder in the short‐wavelength part of the spectrum, however, is likely caused by an increase of absorption of the solution resulting in inner filter effect as shown in Figure S3. It is important to note that the PLE spectrum of the solution with 1 : 130 of PbI_2_ : MAI overlaps with the absorption band of both [PbI_3_]^−^ and [PbI_4_]^2−^ species (Figure 3 (a), Figure S2 (a, b)). To identify the contribution of [PbI_4_]^2−^ species to the PL spectrum, the solution of 0.25 mM of PbI_2_ with the addition of MAI in the ratio of 1 : 130 (PbI_2_ : MAI) was excited with *λ_exc_*=470 nm into the tail of absorption band of [PbI_4_]^2−^ species. The PL spectrum of the solution exhibited the PL peak at 755 nm, with only a slight broadening of the peak in the blue part of the spectrum. However, due to a significant spectral overlap of PL peaks of [PbI_3_]^−^ and [PbI_4_]^2−^ species, the exact deconvolution of the PL spectra is difficult. The comparison between the normalized PL, scaled PLE, and absorption spectra of 0.25 mM of PbI_2_ and 1 : 130 of PbI_2_ : MAI solutions excited with *λ_exc_*=360 and 470 nm respectively is shown in Figure S2 (b).

Based on these observations, the absorption bands related to the presence of lower coordinated [PbI_2_]^0^ solution species are not responsible for the emissive properties of the solutions. On the contrary, the PL peak at 760 nm at low concentrations stems mainly from [PbI_3_]^−^ solution species with a contribution of [PbI_4_]^2−^ species at high concentration of added I^−^.

Interestingly, the solution of pure lead iodide in sub‐millimolar concentrations exhibits luminescence at *λ*=760 nm, while only traces of [PbI_3_]^1−^ can be detected in the absorption spectrum. Thus, the formation of high‐order polyiodide plumbates can be expected in solutions of pure PbI_2_ as well as in the presence of MAI. In Figure 3 (b), the absorption, PLE, and PL spectra of PbI_2_ solutions in DMF are shown for different solution concentrations of 0.1, 1, 10, and 100 mM. The solution containing 0.1 mM of PbI_2_ in DMF showed similar optical properties as the 0.25 mM solution of PbI_2_ (Figure 3 (a)). An increase of the concentration of PbI_2_ to 1 mM did not lead to the appearance of new bands. However, it caused an increase of the absorption of the solution at a wavelength of 380 nm by a factor of 10. This effect is accompanied by a dramatic rise of the intensity of the PLE peak at *λ_max_*=380 nm and corresponding PL peak at *λ_max_*=760 nm. Note that the PL and PLE intensity of the spectra of the 0.1 mM solution were multiplied by 10. A further increase of the concentration of PbI_2_ to 10 mM resulted in an increase of the absorption bands at *λ*=280 and 320 nm. Moreover, a characteristic shoulder at about *λ*=370 nm is observed that can be attributed to the formation of [PbI_3_]^1−^. The increase of the [PbI_3_]^1−^ concentration causes a significant increase of the PLE and PL emission bands. Note that the dramatic increase of the absorption of non‐emissive species ([PbI_2_]^0^) causes parasitic absorption that leads to asymmetric PLE spectra that are cut‐off at their blue edge (see data in Figure 3 (b) for 10 mM PbI_2_). A further increase of the concentration leads to an increase of the absorption tail at wavelengths larger than 400 nm. This is due to the formation of [PbI_4_]^2−^. As parasitic absorption from non‐emissive solution species increases the inner filter effect, thus, only the tail of PLE spectra can be considered in the analysis of spectra evolution.

Characteristic absorption and emission features attributed to different [PbI_2+n_]^n−^ solution species derived from the absorption and emission spectra investigated here are summarized in Table 1 (see Table 1).

**Table 1:** Absorption and emission features of [PbI_2+n_]^n−^ solution species.

	Cryst. PbI_2_	[PbI_2_]^0^	[PbI_3_]^1−^	[PbI_4_]^2−^
*λ_abs_*/nm	494	323	369	420
PLE/nm		n/a	376	n/a^[a]^
PL/nm	505	n/a	755	755^[a]^

^[a]^ peak position overlaps with [PbI_3_]^−^

The experimental data presented above clearly show that lead iodide (PbI_2_) and methylammonium lead iodide (CH_3_NH_3_PbI_3_) perovskite dissolved in DMF have similar absorption and PL spectra (see Figures 1 (b) and (c)). In addition, polyiodide plumbates formed in both solutions exhibited similar decay of photoluminescence (Figure S4). Since the complexes formed in solutions of CH_3_NH_3_PbI_3_ and PbI_2_ show the same optical properties that correlate with the presence of [PbI_3_]^1−^ and [PbI_4_]^2−^, it is apparent that the addition of CH_3_NH_3_I to the PbI_2_/DMSO solution has no influence on the properties of the complexes already formed by PbI_2_ and DMSO. However, drying of the (PbI_2_) and methylammonium lead iodide (CH_3_NH_3_PbI_3_) solutions leads to (CH_3_NH_3_PbI_3_) perovskite and PbI_2_ powders with different absorption and PL spectra (see Figure 1 (a) and (d)). To elucidate the origin of the similarities of the optical properties of PbI_2_ and CH_3_NH_3_PbI_3_ precursor solutions, the chemical equilibrium between different polyiodide plumbate complexes needs to be considered. In solution, the complexes are formed through coordination of Lewis bases such as the solvent molecules to the lead metal center (Pb^2+^) via lone electron pairs on O, N, or S atoms. Instead of simply forming a solvation shell, solvent molecules like GBL, NMP, DMSO, and DMF interact with the precursor species by forming complexes. For Pb^2+^ complexes, coordination numbers (CNs) between 3 and 10 have been experimentally observed.^34^ Since 6 s^2^ shell of Pb^2+^ provides a stereochemically active lone electron pair, these complexes exhibit distortion from idealized geometries and form both „hemidirected” and „holodirected” structures as recently was calculated by E. Radicchi et al.^35^, ^36^ For simplicity, let us assume CN of Pb^2+^ species to be 6 and the Pb^2+^L_6_ complexes to adopt octahedral geometry similar to the PbX_6_ structure of hybrid perovskites.

**Figure 4:**
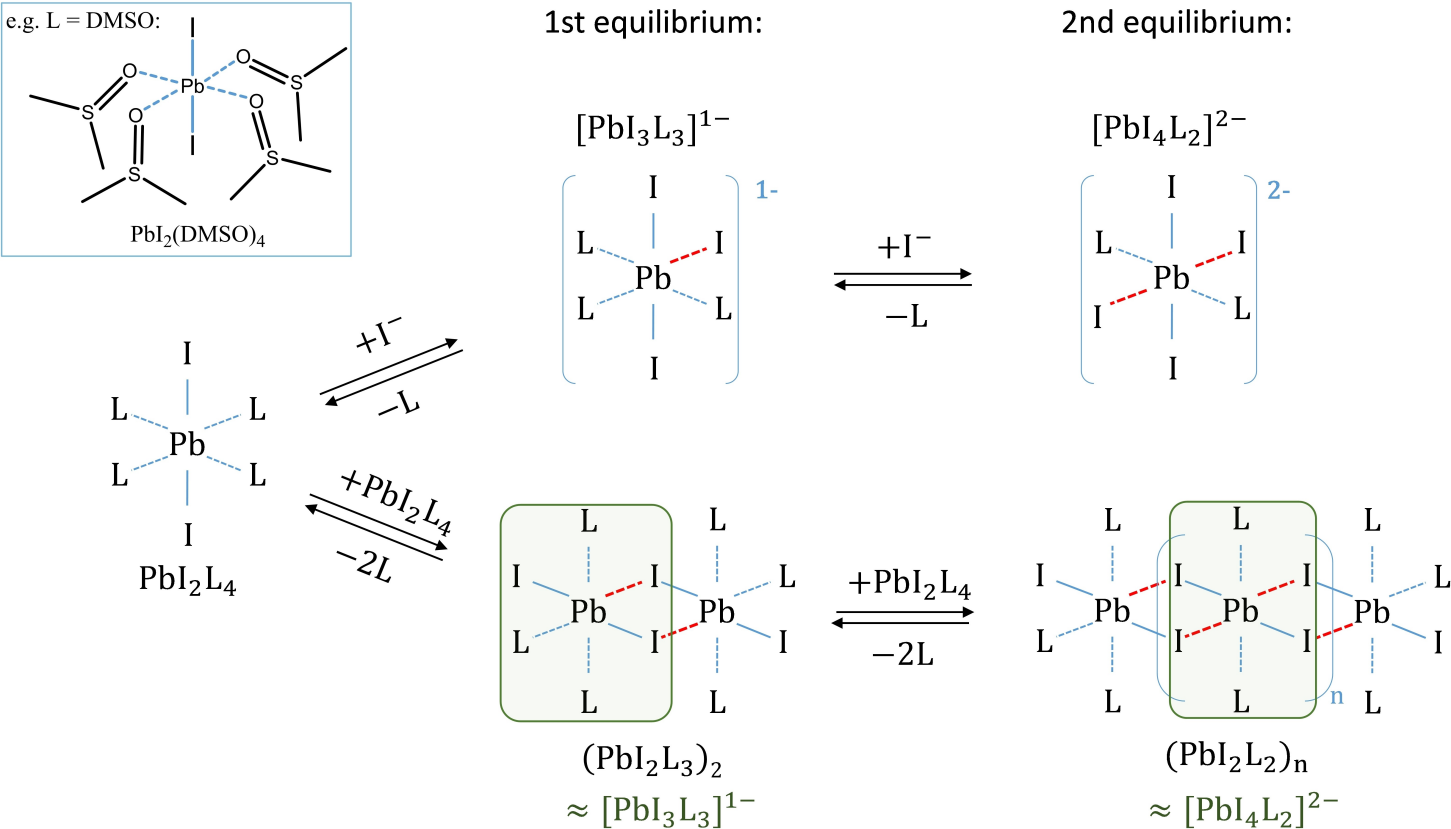
The shift of the chemical equilibrium in a solution of PbI_2_ in form of PbI_2_L_4_ complex by the addition of I^−^ ions (top row) and through the increase of the concentration of PbI_2_ (bottom row). Possible structures of the [PbI_3_L_3_]^−1^ and [PbI_4_L_2_]^2−^ building blocks are indicated by green shaded area.

The chemical equilibrium in the precursor solution can be illustrated starting from dissolved PbI_2_, coordinated by four solvent molecules (L) leading to [PbI_2_L_4_]^0^ (e. g., L=DMSO, [PbI_2_(DMSO_4_)]^0^) as shown on the left side of Figure 4. One commonly described approach to influence the chemical equilibrium and to form different polyiodide plumbates is through the addition of iodide ions (I^−^).^24^ When I^−^ ions are added to the PbI_2_ solution, a competitive binding can be observed between I^−^ ions and solvent molecules (L) towards the Pb^2+^ ions. Even a small amount of I^−^ ions is sufficient to replace solvent molecules and to induce the formation of polyiodide plumbate species [PbI_3_L_3_]^1−^ in the first and [PbI_4_L_2_]^2−^ species in the second equilibrium. This is illustrated at the top of Figure 4. The negative charge on the coordination sphere is compensated by positively charged counter ions such as MA^+^. As a result, the evolution of spectroscopic features of the PbI_2_ solution with increasing MAI content can be attributed to the species [PbI_2_L_4_]^0^, [PbI_3_L_3_]^1−^ and [PbI_4_L_2_]^2−^ (Figure 3(a)).

Since the distinct absorption bands correlated with the presence of the lead‐iodide‐solvent complexes are also observed in the solutions of PbI_2_ (Figure 3 (b)), a similar coordination process is expected between [PbI_2_]^0^ species. With increasing PbI_2_ concentration in the solution, the formation of [PbI_3_]^1−^ and [PbI_4_]^2−^ complexes can be observed, which manifests itself as an increase of intensity of the corresponding absorption bands. In the absence of positively charged methylammonium ions, the charge neutrality of such complexes can be preserved by the interaction with more Pb^2+^ ions through the formation of polymeric polyiodide plumbates (PbI_2_L_x_)_n_. As a result, we propose the formation of polymeric polyiodide plumbates to occur in lead‐iodide precursor solutions at high concentrations, as indicated at the bottom of Figure 4.

Polymeric‐like lead‐halide‐solvent complexes can be formed through the interaction of two or more PbI_2_L_4_ species. In the first equilibrium, coordination between two PbI_2_L_4_ species leads to the formation of dimers (PbI_2_L_3_)_2_. Polymeric (PbI_2_L_2_)_n_ species can be formed when more PbI_2_L_4_ species are added (2^nd^ equilibrium). The exact geometry and electronic structure of the polymeric (PbI_2_L_2_)_n_ species is currently under intense investigation.

Interestingly, dimers (PbI_2_L_3_)_2_ and polymers (PbI_2_L_2_)_n_ contain PbI_3_L_3_ and PbI_4_L_2_ structural motifs, indicated by the green shaded areas in Figure 4 (lower row). This is consistent with the fact that polyiodide plumbates exhibited similar optical properties, whether generated by the addition of I^−^ ions (Figures 3 (a) and 4 (top)) or changing the PbI_2_ concentration (Figures 3 (b) and 4 (bottom)). Consequently, the [PbI_3_]^1−^ and [PbI_4_]^2−^ solution species and PbI_3_L_3_ and PbI_4_L_2_ structural motifs might be causing similar emission bands. Thus, the formation of high‐order polyiodide plumbates and, as a result, hybrid perovskites is predominantly driven by the coordination chemistry of lead and iodide in the solution and is independent of the organic cation. Importantly, the observed PL peak at 760 nm stems from the formation of [PbI_2+n_]^n−^ species.

To unveil the origin of the emission band of species in PbI_2_ and CH_3_NH_3_PbI_3_ solutions, a correlation with the emissive properties of the previously investigated [PbBr_3_]^1−^ and [PbBr_4_]^2−^ species can be made. Oldenburg and Vogler^37^ assigned the emission bands at *λ*=604 nm and 560 nm to [PbBr_3_]^1−^ and [PbBr_4_]^2−^ complexes, respectively, by investigating acetonitrile solutions of tetraethylamine‐lead‐tribromide ((NEt_4_)PbBr_3_) and di‐tetraethylamine‐lead‐tetrabromide ((NEt_4_)_2_PbBr_4_). Yoon et al.^26^ investigated the emissive properties of [PbBr_3_]^1−^ and [PbBr_4_]^2−^ complexes in solution by changing the chemical equilibrium through MABr addition to a 2.7 mM PbBr_2_ solution in DMF. This is similar to the experiments described above (see Figure 3 (a)). Similarly to Oldenburg and Vogler, they attribute the PL emission bands at *λ*=600 nm and 560 nm to [PbBr_3_]^1−^ and [PbBr_4_]^2−^, respectively. The strong agreement between the two studies implies that the excited state of polyhalide plumbates is not affected by solvent and cation and can predominantly be assigned to [PbBr_2+n_]^n−^ complexes. The absorption and emission bands of these species have been attributed to metal‐centered s→p transitions and ligand‐to‐metal charge transfer (LMCT) in halide complexes of s^2^ metals.^37^

Typically, LMCT transitions involve the promotion of an electron from filled p_π_ and p_σ_ orbitals of a ligand (e. g. Br^−^ or I^−^ ions) to unoccupied antibonding p_σ_ orbitals of the metal (here: Pb). The bromide derivatives exhibit mixing of s→p and LMCT transitions, while destabilization of s_σ_ orbitals of the I atom likely leads to an increased contribution of LMCT transition in the emission spectrum of [PbI_2+n_]^n−^. Thus, [PbI_2+n_]^n−^ complexes demonstrate strongly red‐shifted emission with the large Stokes shift. Similarly, large Stokes shifts of several 100 nm have also been observed for [PbCl_3_]^1−^ and [PbCl_4_]^2−^. It has been suggested that larger complex distortion (due to the s^2^ lone pair) of the ground state in comparison to the excited state, where the distortion is no longer favored induces Stokes shift of up to about 200 nm.

Due to the large Stokes shift between absorption and emission bands, polyiodide plumbates demonstrate emissive properties similar to those of the solid‐state CH_3_NH_3_PbI_3_ semiconductor. Intriguingly, in contrast to polybromide and polychloride, polyiodide plumbates actually exhibit emission features that are only about 52 meV higher in energy compared to their solid‐state CH_3_NH_3_PbX_3_ semiconductors (see Table 2). Taking this into consideration, it is possible to assume that the presence of residuals of the emissive [PbI_2+n_]^n−^ species in the solid‐state CH_3_NH_3_PbI_3_ would lead to the formation of defects which strongly resemble optical properties of the solid‐state semiconductor and, thus, do not diminish optoelectronic properties of the material.

**Table 2:** Absorption and emission maxima of CH_3_NH_3_PbX_3_ and [PbX_2+n_]^n−^ solution species reported in the literature and this work

	Iodide, I			Bromide, Br			Chloride, Cl		
	*λ_abs_* [nm]	*λ_emm_* [nm]	*Δλ* [nm]	*λ_abs_* [nm]	*λ_emm_* [nm]	*Δλ* [nm]	*λ_abs_* [nm]	*λ_emm_* [nm]	*Δλ* [nm]
CH_3_NH_3_PbX_3_	765 ^this work^	780 ^this work^	15	520^26^	525^25^	5	398^38^	407^38^	9
PbX_2_	505 ^this work^	505 ^this work^	0	335^39^	379^[39], [d]^	44	272^39^	328^[39], [d]^	56
[PbX_2_]^0^	323 ^[a], this work^ 330^24^	n/a ^[a], this work^		285^26^	n/a		<270^[40], [c]^	n/a	
[PbX_3_]^‐^	367 ^[a], this work^ 370^[24], [a]^ 366^[41], [b]^	755 ^[a], this work^	388	310^[26], [a]^ 306^[37], [b]^	610^[25], [a]^ 604^[37], [b]^	300	273^[42], [c]^	538^[42], [c]^	265
[PbX_4_]^2‐^	423 ^[a], this work^ 425^[24], [a]^ 408^[41], [b]^	755 ^[a,e], this work^	332	360^[26], [a]^ 343^[37], [c]^	560^[26], [a]^560^[37], [c]^	300	294^[42], [c]^	518^[42], [c]^	224

^[a]^ N,N‐dimethyl formamide DMF,^[b]^ acetonitrile (ACN),^[c]^ dimethylsulfoxide (DMSO),^[d]^ at 10 K,^[e]^ peak position overlaps with [PbI_3_]^−^. *Δλ* is the difference between measured positions of maximum intensity of the absorption and emission peaks (Stokes shift).

## 3 Conclusion

In this work, we compare the optical properties of polyiodide plumbate complexes [PbI_2+n_]^n−^ formed in solutions of PbI_2_ as well as CH_3_NH_3_PbI_3_ precursor solutions. The spectral features of [PbI_2+n_]^n−^ solution species were observed in both solutions irrespective of the presence of organic cations or the nature of the polar aprotic solvent. In addition to [PbI_2+n_]^n−^ solution complexes we also propose the formation of polymeric polyiodide plumbate complexes, in particular, in solutions with high concentrations of PbI_2_. For the first time, we show a photoluminescence peak at about 760 nm stemming from the polyiodide plumbate species in solutions of CH_3_NH_3_PbI_3_ and PbI_2_. The PL peak of the [PbI_2+n_]^n−^ solution species is only 52 meV higher in energy than the PL peak observed for CH_3_NH_3_PbI_3_. Consequently, the defects formed by such solution species remaining in the thin films exhibit similar optical properties. Hence, they should not be detrimental to the optoelectronic properties of the solid‐state semiconductor.

## Experimental Section

Lead iodide solutions with different concentrations were prepared by dissolving an appropriate amount of PbI_2_ in dimethyl sulfoxide (DMSO). The solutions were heated at 60 °C and stirred for 12 h before measurements. Solutions of CH_3_NH_3_PbI_3_ precursor solutions were prepared by mixing and dissolving 1 to 1 molar ratio of methylammonium iodide (MAI) and lead iodide in DMSO. Similarly, the solutions were stirred and heated at 60 °C for 12 h before use. Solutions of PbI_2_ in DMF, NMP, GBL, and ACN were prepared using anhydrous solvents as described above. Thin films were deposited by spin‐coating of the precursor solution on a glass substrate at 1000 rpm for 30 s and 5000 rpm for 10 s. At the latest step of the spin‐coating, 100 μL of toluene was dropped on the spinning sample. The same procedure was used for the deposition of PbI_2_ thin films. All precursor solutions and films were prepared in N_2_ atmosphere. The PL spectra in Figure 1 and 2 were excited with a pulsed UV dye laser with excitation wavelength *λ_ex_*=365 nm and 400 nm long‐pass filter. The PL spectra in Figure 3 were excited with *λ_ex_*=300, 320, 380, 395, and 425 nm, *Δλ*=5 nm by means of xenon lamp and double monochromators. To acquire the PLE spectra, the PL intensity was detected at the fixed value (755 nm) as a function of the excitation wavelength.

## Conflict of interest

The authors declare no conflict of interest.

## Supporting information

As a service to our authors and readers, this journal provides supporting information supplied by the authors. Such materials are peer reviewed and may be re‐organized for online delivery, but are not copy‐edited or typeset. Technical support issues arising from supporting information (other than missing files) should be addressed to the authors.

Supplementary
