# Supplementary material for: Building Blocks of Hybrid Perovskites: A Photoluminescence Study of Lead‐Iodide Solution Species
Source: Chemphyschem. 2020 Sep 30;21(20):2327–33. doi: 10.1002/cphc.202000479 (PMC7702157; doi:10.1002/cphc.202000479)
Supplement: Supplementary file 1 — Supplementary [file CPHC-21-2327-s001.pdf]

ChemPhysChem

Supporting Information

# Building Blocks of Hybrid Perovskites: A Photoluminescence Study of Lead-Iodide Solution Species

Oleksandra Shargaieva,* Lena Kuske, Jörg Rappich, Eva Unger,* and Norbert H. Nickel

# Supporting Information

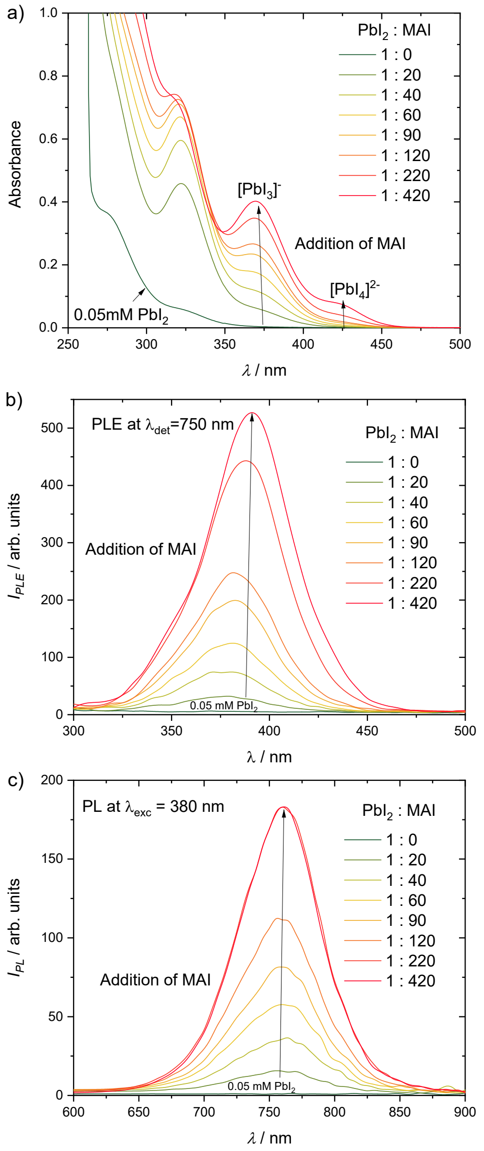


Figure S1. Absorbance (a), photoluminescence excitation (PLE) (b), and photoluminescence emission (PL) (c) spectra of 0.05 mM $PbI_2$ solution in dimethylformamide with addition of methylammonium iodide (MAI). All measurements were performed in 1 cm quartz cuvette.

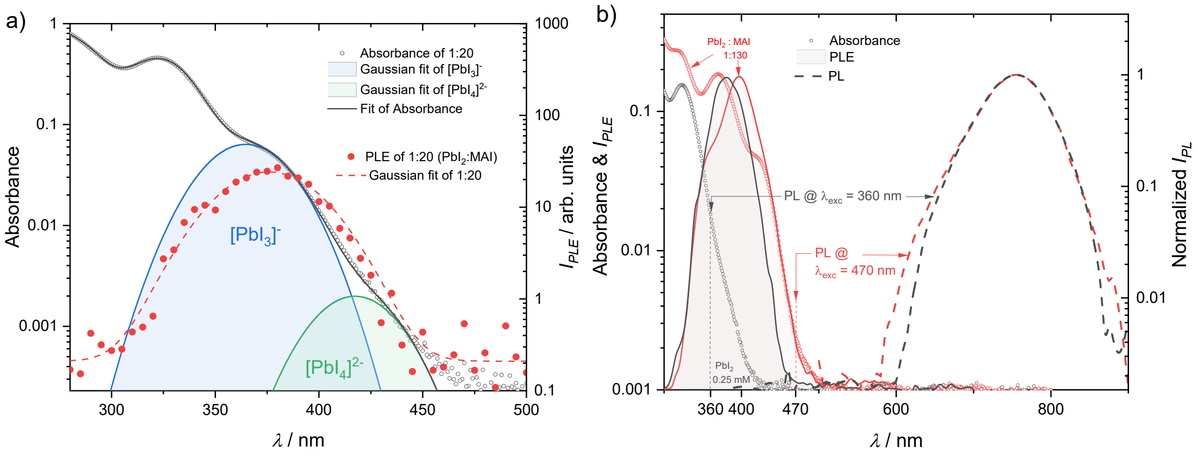


Figure S2. (a) Absorption spectrum (open black symbol) of 0.05 mM $PbI_2$ solution in DMF with addition of MAI in 1:20 ratio of $PbI_2$ : MAI. Fitted curve to the absorption spectrum is shown as a black line; fitted absorption bands of $[PbI_3]^-$ and $[PbI_4]^{2-}$ species are indicated as blue and green shaded areas respectively. The PLE spectrum of the same solution and its Gaussian fit are shown as red symbols and red dotted line respectively. b) Absorption spectra (symbols) of 0.25 mM solution of $PbI_2$ in DMF (black) and solution with addition of MAI in 1:130 ratio of $PbI_2$ : MAI (red). The PL (dashed line) and PLE (shaded area) spectra of corresponding solutions were excited with $\lambda_{exc}$ = 360 and 470 nm and detected at 750 nm respectively. The intensity of PLE spectra was scaled to the absorption band of $[PbI_3]^-$ species.

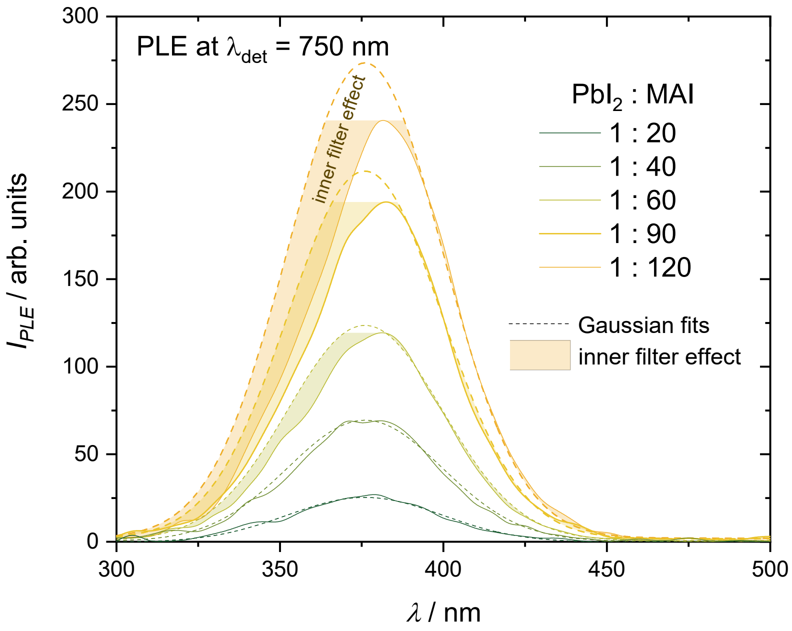


Figure S3. The photoluminescence excitation (PLE) spectra of 0.05 mM $PbI_2$ solution in dimethylformamide with the addition of methylammonium iodide (MAI). The increase of the MAI concentration in the solution leads to a change in the PLE peak shape and position due to inner filter effect caused by the increased absorption of $[PbI_2]^0$ species in solution. The absorption of $[PbI_2]^0$ species affects mainly the short-wavelength part of the spectrum. In order to highlight the influence of inner filter effect, the spectrum of the solution with the addition of MAI in ratio 1:20 of $PbI_2$ : MAI was fitted with Gaussian function. The peak position and peak width obtained from this fit were used for fitting the PLE spectra of solutions with a higher concentration of MAI only considering the long-wavelength part of the spectrum. The difference between the experimental spectra and fitted curves shown as shaded area indicates inner filter effect.

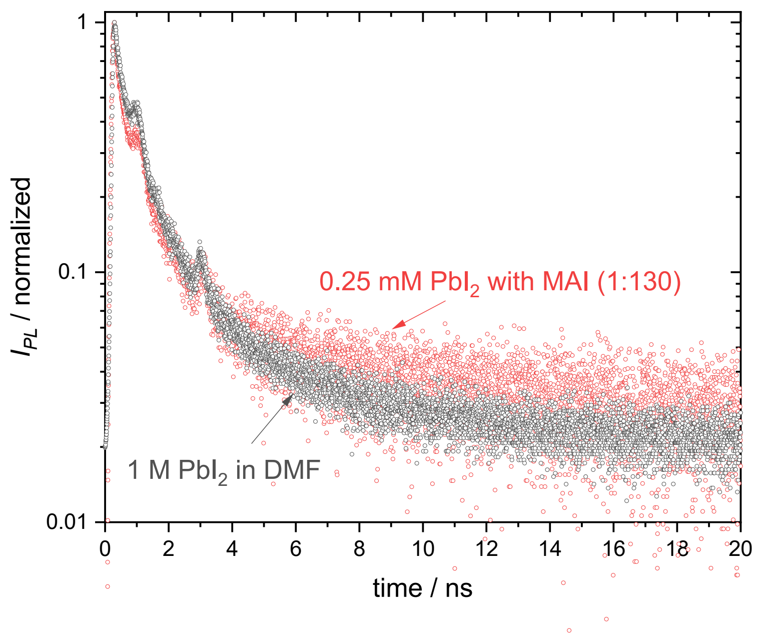


Figure S4. Transient photoluminescence measurements on solutions of 1M $PbI_2$ in DMF (black symbols) and solution of 0.25 mM $PbI_2$ with the addition of MAI in ratio 1:130 ($PbI_2$ : MAI) (red symbols). The PL signal was excited with a pulsed laser with $\lambda_{exc}$ = 490 nm (11.1 MHz repletion rate) and detected at 760 nm.
